# Cancer-derived exosomal HSPC111 promotes colorectal cancer liver metastasis by reprogramming lipid metabolism in cancer-associated fibroblasts

**DOI:** 10.1038/s41419-022-04506-4

**Published:** 2022-01-13

**Authors:** Chong Zhang, Xiang-Yu Wang, Peng Zhang, Tao-Chen He, Jia-Hao Han, Rui Zhang, Jing Lin, Jie Fan, Lu Lu, Wen-Wei Zhu, Hu-Liang Jia, Ju-Bo Zhang, Jin-Hong Chen

**Affiliations:** 1 Department of General Surgery, Huashan Hospital, Fudan University, 12 Wulumuqi Road (M), Shanghai, 200040 China; 2 Institute of Cancer Metastasis, Fudan University, Shanghai, China; 3 Department of Pathology, Huashan Hospital, Fudan University, 12 Wulumuqi Road (M), Shanghai, 200040 China; 4 Department of Infectious Diseases, Huashan Hospital, Fudan University, 12 Wulumuqi Road (M), Shanghai, 200040 China

**Keywords:** Cancer metabolism, Metastasis, Epithelial-mesenchymal transition

## Abstract

Tumor metastasis is a hallmark of cancer. The communication between cancer-derived exosomes and stroma plays an irreplaceable role in facilitating pre-metastatic niche formation and cancer metastasis. However, the mechanisms underlying exosome-mediated pre-metastatic niche formation during colorectal cancer (CRC) liver metastasis remain incompletely understood. Here we identified HSPC111 was the leading upregulated gene in hepatic stellate cells (HSCs) incubated with CRC cell-derived exosomes. In xenograft mouse model, CRC cell-derived exosomal HSPC111 facilitated pre-metastatic niche formation and CRC liver metastases (CRLM). Consistently, CRC patients with liver metastasis had higher level of HSPC111 in serum exosomes, primary tumors and cancer-associated fibroblasts (CAFs) in liver metastasis than those without. Mechanistically, HSPC111 altered lipid metabolism of CAFs by phosphorylating ATP-citrate lyase (ACLY), which upregulated the level of acetyl-CoA. The accumulation of acetyl-CoA further promoted CXCL5 expression and secretion by increasing H3K27 acetylation in CAFs. Moreover, CXCL5-CXCR2 axis reinforced exosomal HSPC111 excretion from CRC cells and promoted liver metastasis. These results uncovered that CRC cell-derived exosomal HSPC111 promotes pre-metastatic niche formation and CRLM via reprogramming lipid metabolism in CAFs, and implicate HSPC111 may be a potential therapeutic target for preventing CRLM.

## Introduction

Colorectal cancer (CRC) is the third most common malignancy and has risen to the second leading cause of cancer-related death worldwide [1]. Metastasis is the major cause of CRC-related death, and the liver is the most common organ for CRC metastasis [2, 3]. Although surgical techniques and systemic therapies are advanced, the prognosis of CRC liver metastasis (CRLM) patients remains poor. It is urgent to explore the underlying mechanism and exploit effective therapeutic strategies for CRLM patients.

Prior to metastatic colonization, primary cancer cells are able to influence the microenvironment of distant organs by facilitating the formation of pre-metastatic niche [4–6]. Cancer-associated fibroblasts (CAFs), an activated sub-population of fibroblasts, are one of the main components of stromal cells in the pre-metastatic niche and exhibit distinct tumorigenic properties [7, 8]. Recent studies demonstrated that CAFs secrete chemokines, growth factors, extracellular matrix and matrix metalloproteinases to regulate tumor initiation, progression and metastasis [9, 10]. Exosomes, ranging from 50–160 nm in diameter, are cancer-derived vesicles to participate in pre-metastatic niche formation by deliver tumor products including proteins, nucleic acids and lipid to distant organs, and can transform fibroblasts to CAFs [11, 12]. However, the mechanisms underlying activation of fibroblasts by cancer-derived exosomes to promote metastatic colonization in CRLM deserves further investigation.

Metabolic reprogramming is one of the essential hallmarks of cancer cells. The metabolic adaptation of cancer cells is necessary for their proliferation, adaption to tumor microenvironment and distant metastasis [13, 14]. In our previous study [15], we revealed that CRC cells show specific metabolic reprogramming during the process of liver metastasis. Recently, accumulating evidence indicate that metabolic adaptation also exists in non-tumor stromal cells, whose activation participates in the process of tumor metastasis [16]. However, most studies have focused on the metabolic changes of CAFs resident in the primary tumor [17, 18], and there remains little known about the mechanism of metabolic reprogramming of CAFs in the pre-metastatic niche during the process of CRC liver metastasis.

In this study, we uncovered the critical role of CRC cell-derived exosomal HSPC111 in facilitating pre-metastatic niche formation by inducing lipid metabolic reprogramming of CAFs in liver. Importantly, the expression level of HSPC111 was detected at higher level in serum exosomes, primary tumors and CAFs of CRC patients with liver metastasis than those without. Our studies revealed a previously unknown mechanism on liver metastasis of CRC, providing a potential therapeutic strategy to target CRLM.

## Materials and methods

### Cell lines

The human colorectal cancer cell line HCT116 was purchased from the China Center for Type Culture Collection (Wuhan, China); human colorectal cancer cell lines HT29 and SW480 were purchased from the American Type Culture Collection (Rockville, MD, USA); and the HEK293T cell line, human colorectal cancer cell line SW620 and human hepatic stellate cell line LX-2 were purchased from Cell Bank of Type Culture Collection of the Chinese Academy of Sciences (Shanghai Institute of Cell Biology). All cell lines were cultured in DMEM (Gibco) supplemented with 10% FBS (Gibco) and 1% penicillin and streptomycin (Beyotime Biotech Co. Ltd., China) at 37 °C in 5% CO_2_. Cell lines were authenticated by short tandem repeats profiling and confirmed to be mycoplasma negative.

### Exosome isolation and characterization

Exosomes were isolated from cell culture supernatants by using ExoQuick-TC Exosome Isolation kit (System Biosciences, Palo Alto, USA) [19] and from serum by using ExoQuick exosome precipitation solution (System Biosciences, Palo Alto, USA) [20] as described previously, respectively. Briefly, the culture supernatant was collected and centrifuged at 4000 × *g* for 30 min to remove cell debris, and one-fifth of ExoQuick-TC was added to supernatant following by incubating overnight at 4 °C. Exosomes were isolated by centrifugation at 1500 × *g* for 30 min. Serum was harvested and centrifuged at 500 × *g* and 10,000 × *g* for 10 min to remove intact cells and cell debris, individually. 1 mL serum was mixed with 250 μL of solution and incubated for 30 min at 4 °C, and then centrifuged at 1500 × *g* for 30 min to isolate exosomes. Each pellet was resuspended with PBS and the protein concentration of exosome was determined by BCA assay (TAKARA, Japan). The characterization of exosomes was analyzed by transmission electron microscopy (TEM) (FEI, Tecnai F20). The density and size of exosomes were tracked by nanosight particle tracking analysis (NTA) (NS300 system, NanoSight technology, Malver, UK).

### Exosome labeling and tracing

To assess transfer of exosomes into LX-2, CRC cell-derived exosomes were labeled with DiO dye (Rengen Biosciences, China) according to the manufacturer’s instructions. Briefly, exosomes were incubated with DiO dye for 30 min at 37 °C. Excessive DiO was removed with Spin Column. Dio-labeled exosomes were added to the culture media of LX-2 cells and incubated for 8 h at 37 °C. After stained with DAPI (Boster Biological Technology co., Ltd, China), LX-2 cells were observed with confocal microscopy (Leica, Germany).

For exosome tracking experiments in vivo, purified exosomes (50 μg/mice) were labeled by using DiR dye (KeyGEN BioTECH, China) and injected into BALB/c mice via the tail vein. As a control, mice were injected with the same volume of PBS. IVIS imaging system (PerkinElmer) was used for tracking exosome distribution in vivo. To evaluate exosome uptake by liver, all mice were sacrificed after 24 h, and the liver tissues were harvested for immunofluorescence and photographed by using a confocal microscopy.

### Animal model induction and treatment

In each animal experiment, mice were randomly allocated to each group. All animal experiments were performed in accordance with protocols approved by the institutional review board of Department of Laboratory Animal Science, Fudan University, and conformed to the National Guidelines for Animal Usage in Research. All animal experiments were performed on 5–7 weeks male nude mice (BALB/c nu/nu) (SLAC Laboratory Animal Co., Ltd, Shanghai, China).

To evaluate the roles of exosomes in CAFs in CRLM, the mice were injected with exosomes (10 μg in 100 μL PBS) derived from HCT116 cells via tail vein every other day for 3 weeks. Subsequently, HCT116 cells were injected into the spleens of the mice to induce liver metastasis model. Mice were also treated with exosomes via tail vein injection twice a week continuously. Four weeks after HCT116 cells injection, all mice were euthanized and liver tissues were collected for the isolation of primary fibroblasts.

For analyzing the roles of exosomal HSPC111 in CRLM, mice were treated with exosomes (10 μg in 100 μL PBS) via tail vein injection every other day for 3 weeks. The same volume of PBS was injected into mice in control groups. Luciferase-labeled HCT116 cells and SW480 cells were used to induce liver metastasis model, which evaluated by using IVIS Lumina LT series III (PerkinElmer, MA, USA). For studies of CXCR2 inhibitor in vivo, mice were treated with 50 mg/kg navarixin (CXCR2 inhibitor) (MCE, China) or vehicle (1% methyl cellulose) orally once daily for 4 weeks. Four weeks after CRC cells injection, mice were euthanized and liver metastasis were evaluated by HE staining.

### Isolation and culture of primary fibroblasts

For the isolation of human primary liver fibroblasts, paired fresh CRLM and distant non-tumor liver tissues were obtained from CRLM patients and normal liver tissues were obtained from hepatic hemangioma patients underwent partial liver resection at Department of General Surgery, Huashan hospital, Fudan University (Shanghai, China). None of the patients had received radiotherapy or chemotherapy prior to surgery. The specimens were used after obtaining written, informed consent from the patients and with the approval of the Ethical Committee of Huashan hospital, Fudan University (Shanghai, China). Tissues were finely minced with scalpel and enzymatically dissociated in 1 mg/mL collagenase IV (Solarbio^®^ Life Sciences, China) at 37 °C for 2 h with frequent shaking. The cell suspensions were filtered and centrifuged at 300 g for 5 min and then resuspended in DMEM with 10% FBS at 37 °C in 5% CO_2_. After incubating for 24 h, the fibroblasts were attached to the culture plate, and the unattached other cells were removed.

### Immunofluorescence

For cell immunofluorescence, the cells were fixed with 4% paraformaldehyde for 15 min and permeabilized in 0.1% Triton X-100 for 20 min. After being blocked with 5% bovine serum albumin, the cells were incubated with primary antibodies overnight at 4 °C. Then the cells were incubated with secondary antibody for 1 h at room temperature. For tissue immunofluorescence, tissues were fixed in a mix of 2% paraformaldehyde and 20% sucrose solution for 24 h at room temperature, followed by embedding and section. Nuclei were stained with DAPI. Fluorescent images were taken with a fluorescent microscope. Information of the antibodies are listed in Supplementary Table 1.

### Metabolomic analysis

LX-2 cells (1 × 10^5^ cells) were treated with 100 μg exosomes derived from CRC cells and incubated for 48 h. Then the LX-2 cells were frozen in liquid nitrogen for quenching metabolism. To analyze metabolomics, samples were analyzed by Omicsolution Co., Ltd (Shanghai, China). Variable importance in projection (VIP) was used to measure the different expression of metabolite in each group, and VIP values >1 were considered as differential expression. All differentially expressed metabolites were selected for heat map and enrichment analysis.

### Proteomic analysis

LX-2 cells were cultured with HCT116-derived exosomes for 48 h. Then the cells were collected for proteomics analysis. Isobaric tags for relative and absolute quantification (iTRAQ)-based proteomic analysis was conducted by Omicsolution Co., Ltd (Shanghai, China). The functions of the multiple protein contents were obtained by searching the UniProt database. Proteins were filtered by using the following thresholds: p value < 0.05 and 1.2-fold change. All differentially expressed proteins were selected for heat map and Kyoto Encyclopedia of Genes and Genomes (KEGG) enrichment analysis.

### Lentiviral transduction

The short hairpin RNA (shRNA) targeting HSPC111 (shHSPC111 #1, shHSPC111 #2), shRNA targeting ACLY (shACLY) and negative control (shCtrl #1, shCtrl #2) were inserted into the lentiviral vector produced by Hanbio Tech (Shanghai, China). HCT116 cells were transfected with lentivirus (pHBLV-U6-MCS-CMV-ZsGreen-PGK-PURO) targeting HSPC111 and LX-2 cells were transfected with lentivirus (pHBLV-U6-MCS-CMV-ZsGreen-PGK-PURO) targeting ACLY at a multiplicity of infection (MOI) of 20. The sequences of shRNAs are presented in Supplementary Table 2.

Lentiviral vectors that overexpress full-length human HSPC111 (pHBLV-CMV-MCS-6x-His-HSPC111-EF1-ZsGreen-T2A-puro) or ACLY (pHBLV-CMV-MCS-3x-Flag-ACLY-EF1-ZsGreen-T2A-puro), and negative control (pHBLV-CMV-MCS-EF1-ZsGreen-T2A-puro) were purchased from Hanbio Tech (Shanghai, China). The overexpressed lentiviruses targeting HSPC111 were transfected with SW480 cells and those targeting ACLY were transfected with LX-2 at a MOI of 20. All stable cell lines were obtained by 6 μg/mL puromycin (Hanbio Tech) selection for 1 weeks followed by subsequent assays.

### Clinical specimens

A total of 66 human non-metastatic primary CRC tissues (NMCT) and paired peritumoral normal colorectal tissues (NCT), and 136 human primary CRC tissues with liver metastasis (CTLM) were collected between 2014 and 2019 from Department of General Surgery, Huashan hospital, Fudan University (Shanghai, China). Serum samples were collected from these patients and healthy people. All procedures were performed with the approval of the Ethical Committee of Huashan hospital, Fudan University (Shanghai, China). Patient consent was obtained before the start of the study. The clinical information of all patients was included in Supplementary Table 3.

### Tissue microarray (TMA)

Representative tumor areas were microscopically selected on HE stained sections. Tissue cores were punched from the defined regions on the donor block by using 2-mm punch needles and inserted into the receiver block followed by histological sectioning and mounting onto slides.

### Immunohistochemistry (IHC)

Sections were taken from the TMA paraffin blocks, deparaffinized in xylene and rehydrated in a descending series of alcohol. After antigen retrieval by using citrate buffer, the sections were incubated in 3% H_2_O_2_ to block endogenous peroxidase activity. Then the sections were incubated with primary antibody for 1 h at room temperature. After incubated with a horseradish peroxidase (HRP)-labeled polymer and 3,3-diaminobenzidine chromogen solution (Dako EnVision), the sections were counterstained with Harris hematoxylin (Solarbio^®^ Life Sciences, China). The immunohistochemical analysis of HSPC111 in the TMA sections was performed using a semiquantitative scoring system ranging from 0 to 3 points. The presence of cytoplasmic staining was considered positive, and according to the staining intensity, the immunopositivity was graded as none (0), weak (1), moderate (2), and strong (3).

### Acetyl-CoA and citrate measurement

The concentration of acetyl-CoA was detected by using Acetyl-CoA assay kit (Sigma MAK039) according to manufacturer’s protocols. Briefly, cells were harvested in RIPA buffer (Beyotime Biotech Co. Ltd., China). The cellular protein was precipitated with PCA (BioVision K808–200). The supernatant was neutralized by potassium bicarbonate and then measured by using fluorescence assay method (λ_ex_ = 535/ λ_em_ = 587 nm). The concentration of citrate was measured by using citrate colorimetric assay kit (BioVision K655–100) according to manufacturer’s protocols. Briefly, cells were rapidly homogenized with citrate assay buffer, then centrifuged at 15,000 × g for 10 min to remove cell debris. The cellular protein was precipitated with PCA and optical density (OD) value was measured at 570 nm.

### RNA sequencing (RNA-seq)

The total RNA of the LX-2 cells incubated with appropriate exosomes for 48 h in vitro was extracted using TRIzol reagent (Invitrogen) and subjected to Biomarker Technologies Corporation (Beijing, China) for RNA sequencing. All usable reads were uniquely mapped to a gene that was used to evaluate the expression level. The DEGseq R package was used to analyze differentially expressed genes in two samples based on the conditions of a fold change (FC) ≥ 1.5 and *p* values < 0.05. All differentially expressed genes were used for heat map analysis.

### Co-immunoprecipitation (Co-IP)

LX-2 cells were lysed in NP-40 lysis buffer (Solarbio^®^ Life Sciences, China) supplemented with protease inhibitor cocktail (PIC) (Solarbio^®^ Life Sciences, China) for 30 min on ice. The lysates were clarified by centrifugation at 12,000 rpm for 15 min at 4 °C, and then incubated with antibodies (Supplementary Table 1) for overnight at 4 °C followed by mixing with protein A/G magnetic beads (MCE, China). HEK293T cells were co-transfected with His-HSPC111 and Flag-ACLY lentivirus were lysed with NP-40 lysis buffer. Then the cell lysate was mixed with His antibody or Flag antibody for overnight at 4 °C followed by incubation with Anti-His Magnetic Beads (Sango Biotech, China) and Anti-Flag Magnetic Beads (MCE, China), respectively. Immunocomplexes were washed and boiled for western blot analysis.

### Glutathione-s-transferase (GST) pull-down assays

GST pull-down assays was performed as described previously [21]. Briefly, the pGEX-6p-1 and pGEX-GST-HSPC111 plasmid were transformed into BL21 competent cells (Thermo Fisher Scientific, EC0114) to obtain GST and GST-HSPC111 proteins, respectively. The pCMV-Flag-ACLY plasmid was transfected into 293T cells to express ACLY protein. The Pierce^TM^ GST Protein Interaction Pull-Down Kit (Thermo Fisher Scientific, 21516) was used according to the manufacturer’s instructions. BL21 cells containing GST or GST-HSPC111 proteins were treated with pull-down lysis buffer and immobilized on equilibrated glutathione agarose resin at 4 °C for 2 h. The resin was washed with wash solution (TBS with Pull-down lysis buffer), and 293T lysates containing Flag-ACLY protein were added, followed by incubation at 4 °C for 12 h. After washing with wash solution, the resin was eluted with glutathione elution buffer. The protein samples were measured by western blot.

### Chromatin immunoprecipitation (ChIP)

ChIP was performed using a SimpleChIP^®^ Enzymatic Chromatin IP kit (Cell Signaling Technology, 9003) according to the manufacturer’s instructions. Briefly, crosslinking of proteins-DNA was carried out using 37% formaldehyde for 10 min at room temperature. The crosslinking was quenched by adding glycine and cells were scraped into 1 mM PIC. The suspension was centrifuged and the pellet was resuspended in buffer A + DTT + PIC and buffer B + DTT containing micrococcal nuclease. The nuclei pellet was further lysed by sonication, and the supernatant was incubated with H3K27ac antibody overnight at 4 °C with rotation. The samples then incubated with protein G magnetic beads, and DNA was eluted from the protein G magnetic beads by ChIP Elution Buffer. qRT-PCR was performed using SimpleChIP^®^ Universal qPCR Master Mix (Cell Signaling Technology, 88989) to detect eluted DNA. The primer sets are listed in Supplementary Table 4.

### Western blot analysis

Western blot was performed as described previously [22]. Briefly, the cells or exosomes were lysed in RIPA lysis buffer. Protein concentrations were detected by BCA assay. Protein was separated by 10% or 15% SDS-PAGE and then transferred to nitrocellulose membranes (Millipore, USA). After incubated with primary antibody overnight at 4 °C, the membranes were incubated with HRP-conjugated secondary antibody. The protein complex was detected using enhanced chemiluminescence reagents (Millipore, USA). Information of the antibodies are listed in Supplementary Table 1.

### Quantitative real-time PCR (qRT-PCR)

qRT-PCR was performed as described previously [22]. Briefly, total RNA was extracted from cells with TRIzol reagent (Life Technologies, USA) according to the manufacturer’s instructions. The concentration of the RNA was determined using NanoDrop 2000 (Thermo Fisher Scientific). The cDNA was synthesized with the PrimeScript RT Master Mix (Takara, Japan). qRT-PCR was performed using TB Green Premix Ex Taq (Takara, Japan) on an ABI PRISM 7900 Sequence Detection System (Applied Biosystems, Carlsbad, USA). The primer sets for qRT-PCR are listed in Supplementary Table 4.

### Conditioned medium (CM) preparation

LX-2 cells were cultured with appropriate exosomes (20 μg/mL) from CRC cells for 48 h. Medium was collected and centrifuged at 300 × *g* for 10 min to remove the cells, at 2000 × *g* for 10 min to remove the dead cells and at 10,000 × *g* for 30 min to remove the cell debris. Then, the supernatant was filtered through a 0.025 μm filter (Millipore, USA) to remove exosomes and stored at −80 °C for further studies.

### Enzyme-linked immunosorbent assay (ELISA)

Cells were cultured in the presence of appropriate exosomes (20 μg/mL) for 48 h before supernatants were collected, and the CXCL5 level was detected by CXCL5 ELISA kit (EK0728, BOSTER) according to the manufacturer’s protocols.

### Cell migration assay

HCT116 cells or SW480 cells (2.5 × 10^5^/well) were plated on the upper part of a 24-well transwell chamber (Corning, NY, USA) in 200 μL specific CM. The medium in the lower chamber was supplemented with DMEM containing 10% FBS. After 24 h, cells that migrated to the underside of the membrane were fixed using methanol, stained with 0.1% crystal violet, and imaged and counted with a microscope (Leica, Wetzlar, Germany).

### Wound-healing assay

HCT116 cells or SW480 cells (1 × 10^6^/well) were plated into 6-well plates in 2 mL 10% DMEM medium. After cells were grown to a confluent layer, the cell monolayers were scratched using a pipette tip. The scratched cells were removed by gently washing with non-FBS DMEM gently. Then the cells were cultured with specific CM for 48 h. Images were obtained at time point 0 and 48 h.

### Statistical analysis

All the data were presented as mean ± standard deviation of the mean (SD). SPSS software version 22.0 was used for statistical analyses. The differences between groups were assessed using the Student’s *t* test and χ^2^ test. A *p* value of <0.05 was considered statistically significant.

## Results

### CRC cell-derived exosomal HSPC111 promotes CAFs activation

**Fig. 1:**
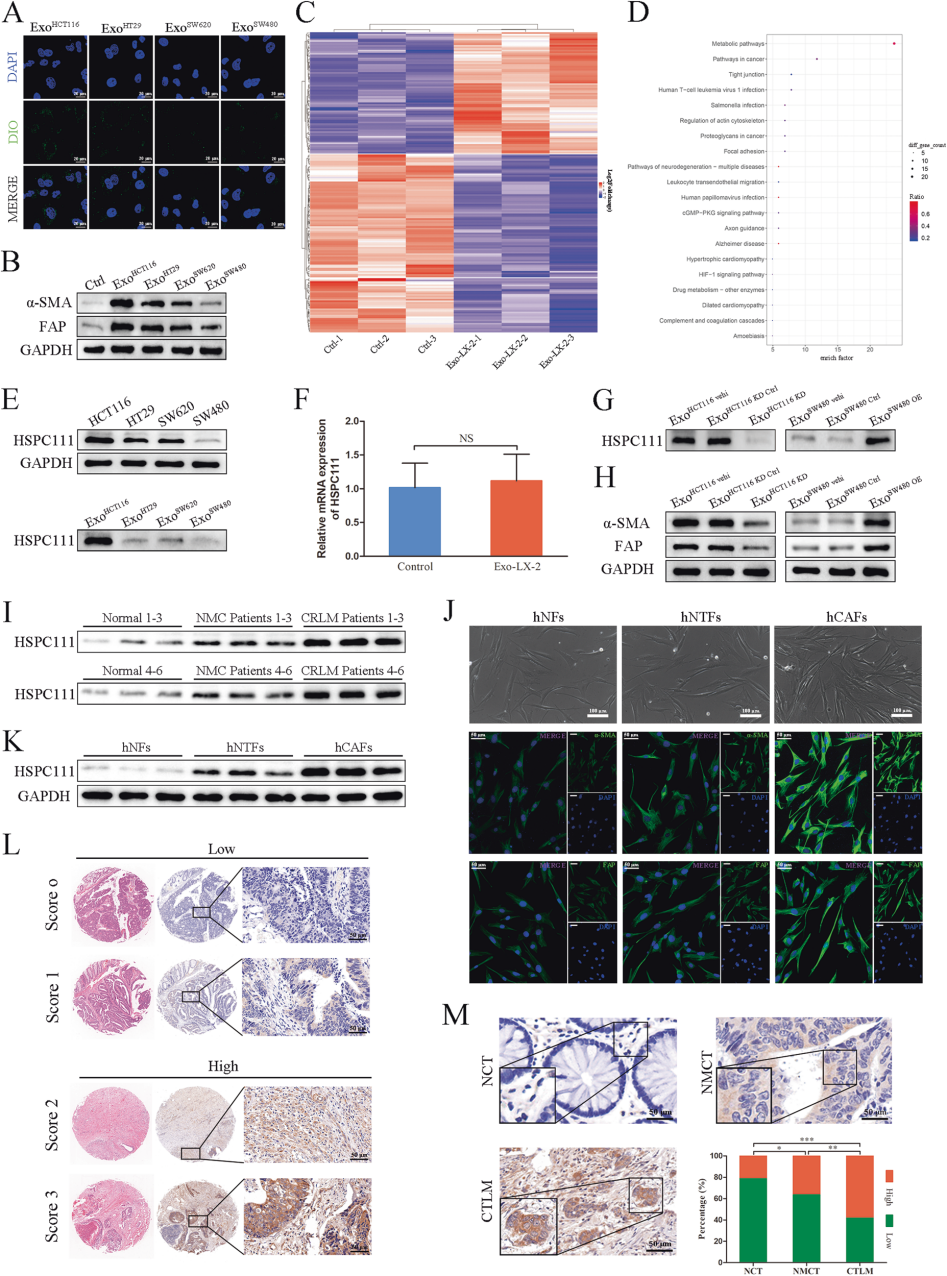
CRC cell-derived exosomal HSPC111 promotes CAFs activation and correlates with liver metastasis in CRC patients. **A** Uptake of DiO-labeled exosomes (green) was analyzed by immunofluorescence microscopy. Scale bar = 20 μm. **B** Western blot showed HSCs were activated by CRC cell-derived exosomes. **C** Differentially expressed proteins identified by iTRAQ in LX-2 cells incubated with Exo^HCT116^. **D** Bubble diagram of KEGG pathway enrichment analysis related to LX-2 cells incubated with Exo^HCT116^. **E** Western blot showed protein level of HSPC111 in different CRC cells and their derived exosomes. **F** qRT-PCR showed mRNA level of HSPC111 in CAFs have no significant difference compared with control group. **G** Western blot showed protein level of HSPC111 in different CRC cell-derived exosomes. **H** Western blot showed exosomal HSPC111 induces the activation of CAFs. **I** Western blot showed protein level of HSPC111 in serum exosomes isolated from patients and healthy volunteers (*n* = 6). **J** Normal fibroblasts (hNFs), non-tumoral fibroblasts (hNTFs) and cancer-associated fibroblasts (hCAFs) isolated from patient liver tissues were photographed by light micrographs (top). Scale bar = 100 μm. Representative immunofluorescence staining of α-SMA and FAP in hNFs, hNTFs and hCAFs isolated from patient liver tissues (bottom). Scale bar = 50 μm. **K** Western blot showed protein level of HSPC111 in hNFs, hNTFs and hCAFs isolated from patient liver tissues (*n* = 6). **L** Scores indicate HSPC111 level in representative CRC tissues. Low group: score 0–1; high group: score 2–3. **M** Representative images of immunohistochemical staining and quantification of HSPC111expression in NCT, NMCT and CTLM specimens. Scale bar = 50 μm. Each experiment was performed in triplicate. Data are shown as mean ± SD. *P < 0.05, **P < 0.01, ***P < 0.001, ns not significant.

Four CRC cell lines with different migration and invasion abilities were chosen, among which HCT116 and SW620 cells were high-metastatic tumor cells, versus HT29 and SW480 cells which were low-metastatic tumor cells, and exosomes were isolated from supernatant of CRC cells. The morphology, size and characteristic protein of exosomes were confirmed by TEM, NTA and western blot, respectively (Supplementary Fig. 1A, B). To evaluate the delivery efficacy of exosomes, we labeled exosomes with Dio. Immunofluorescence imaging indicated the presence of Dio spots in recipient HSCs, suggesting that labeled exosomes were delivered to HSCs (Fig. 1A).

To compare the abilities of educating HSCs into CAFs among the four CRC cell-derived exosomes, the expression of α-SMA and FAP in treated HSCs were evaluated by western blot, which showed most highly in LX-2 cells incubated with HCT116-derived exosomes (Exo^HCT116^) (Fig. 1B). Meanwhile, the shape of LX-2 cells incubated with Exo^HCT116^ showed fibroblast-like appearances (spindle-like and spread) (Supplementary Fig. 1C). To investigate the molecular mechanism responsible for promoting CAFs activation at the protein level, we conducted the iTRAQ proteomic analysis on LX-2 cells incubated with Exo^HCT116^ and control. The heat map of differentially expressed proteins was shown in Fig. 1C (Supplementary Table 5). Meanwhile, the KEGG pathway enrichment analysis indicated that metabolic pathways were the most highly represented pathway (Fig. 1D). Among these differentially expressed proteins, HSPC111 was the most markedly elevated in LX-2 cells following Exo^HCT116^ incubation. Furthermore, western blot indicated that the expression of HSPC111 was higher in HCT116 cells and lower in SW480 cells, which is consistent with their derived exosomes (Fig. 1E). Intriguingly, qRT-PCR showed that HSPC111 mRNA have no significant change in LX-2 cells incubated with Exo^HCT116^ compared with control (Fig. 1F), which indicated that the elevated HSPC111 protein in LX-2 cells was delivered by CRC cell-derived exosomes.

Then we generated two HSPC111-specific shRNAs to silence the HSPC111 expression. shRNA#1, which induced more significant knockdown effect, was selected for knocking down HSPC111 expression in HCT116 cells (HCT116 KD) that highly express HSPC111 (Supplementary Fig. 2A). Meanwhile, HSPC111 was overexpressed in SW480 cells (SW480 OE) that minimally express HSPC111 (Supplementary Fig. 2B). Subsequently, we found that the protein level of HSPC111 was decreased in HCT116 KD cell-derived exosomes (Exo^HCT116 KD^) and increased in SW480 OE cell-derived exosomes (Exo^SW480 OE^) (Fig. 1G). We next investigated the effect of exosomal HSPC111 on CAFs activation. Western blot showed that CAFs were activated following incubated with Exo^SW480 OE^, which was abolished by incubating with Exo^HCT116 KD^ (Fig. 1H). These findings demonstrated that CRC cell-derived exosomal HSPC111 induces the activation of CAFs.

### HSPC111 correlates with liver metastasis in CRC patients

To investigate whether HSPC111 in serum exosomes correlates with CRLM, exosomes from the serum of non-metastasis CRC patients, CRLM patients and healthy volunteers (normal) were isolated (Supplementary Fig. 1D, E). Western blot showed that the protein level of HSPC111 was higher in the serum exosomes from CRLM patients than non-metastasis CRC patients and normal (Fig. 1I).

To evaluate the protein level of HSPC111 in CAFs and normal fibroblasts, we isolated primary fibroblasts from paired liver metastases (hCAFs) and distant non-tumor liver tissues (hNTFs) from the same patients, and normal liver tissues (hNFs) from hemangioma patients (Fig. 1J). The presence of other cell types including endothelial cells, epithelial cells and immune cells were excluded by staining with the corresponding makers CD31, EpCAM and CD45 (Supplementary Fig. 3A). Immunofluorescence staining confirmed that α-SMA and FAP levels were higher in hCAFs and hNTFs than those in hNFs (Fig. 1J). We then examined the protein level of HSPC111 in these primary fibroblasts, and found that HSPC111 levels were increased in hCAFs and hNTFs compared with hNFs (Fig. 1K).

Meanwhile, to investigate whether HSPC111 in CRC tissues is associated with CRLM, we evaluated HSPC111 expression by IHC in human NMCT, NCT and CTLM tissues. These patients were divided into low and high HSPC111 expression group according to the immunostaining scores (Fig. 1L). Compared to the NCT and NMCT, HSPC111 level significantly increased in CTLM (Fig. 1M). These results demonstrated that HSPC111 in serum exosomes and CRC tissues correlates with CRLM.

### CRC cell-derived exosomal HSPC111 promotes CRLM in vivo

**Fig. 2:**
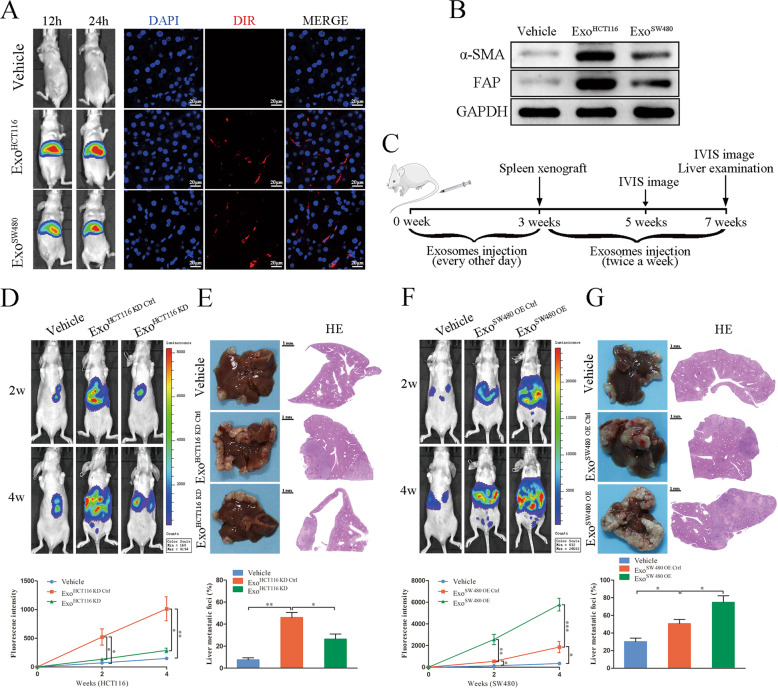
CRC cell-derived exosomal HSPC111 promotes CRLM in vivo. **A** Representative images of mouse received intravenous injection of DiR-labeled HCT116 and SW480 cells-derived exosomes (left) and confocal microscopy of DiR-labeled exosomes in liver tissues (right). Scale bar = 20 μm. **B** The effects of Exo^HCT116^ and Exo^SW480^ on the activation of HSCs in mouse liver primary fibroblasts was analyzed by western blot. **C** Experimental scheme in exosomes and CRC cells induced CRLM mice model. **D** IVIS imaging on experimental liver metastasis of indicated mice treated with Exo^HCT116 KD^ and their relative control. Luciferase-labeled HCT116 cells were used to perform experimental liver metastasis model (*n* = 6). **E** Representative pictures and quantitative results of hematoxylin and eosin (H&E) staining of liver tissue sections from indicated mice. Scale bar = 1 mm. **F** IVIS imaging on experimental liver metastasis of indicated mice treated with Exo^SW480 OE^ and their relative control. Luciferase-labeled SW480 cells were used to perform experimental liver metastasis model (*n* = 6). **G** Representative pictures and quantitative results of HE staining of liver tissue sections from indicated mice. Scale bar = 1 mm. Each experiment was performed in triplicate. Data are shown as mean ± SD. *P < 0.05, **P < 0.01, ***P < 0.001.

To evaluate the distribution of exosomes in vivo, DiR-labeled exosomes were intravenously injected into mice. In vivo tracing assay showed that CRC cell-derived exosomes accumulated in liver, which could be also confirmed by immunofluorescence (Fig. 2A). Importantly, western blot showed that the levels of α-SMA and FAP were increased in exosomes injection group, compared with vehicle group, which indicated that the activation of CAFs in liver tissues after exosome injection (Fig. 2B). To further investigate whether exosomal HSPC111 affects CRLM in vivo, mice were treated with exosomes containing different levels of HSPC111, following by intrasplenic injection of luciferase-labeled HCT116 and SW480 cells as experimental metastasis models (Fig. 2C). In vivo imaging assay showed that purified HSPC111-enriched exosomes promoted liver metastasis, whereas silencing HSPC111 in exosomes markedly reduced the liver metastatic growth (Fig. 2D, F). Histological analysis further confirmed the metastatic promoting effect by HSPC111-enriched exosomes and silencing HSPC111 in exosomes decreased liver metastasis (Fig. 2E, G). Taken together, these results demonstrated that exosomal HSPC111 mediated the pre-metastatic role of CRC cell-derived exosomes during the process of CRLM.

### CRC cell-derived exosomal HSPC111 alters lipid metabolism by increasing the level of acetyl-CoA in CAFs

**Fig. 3:**
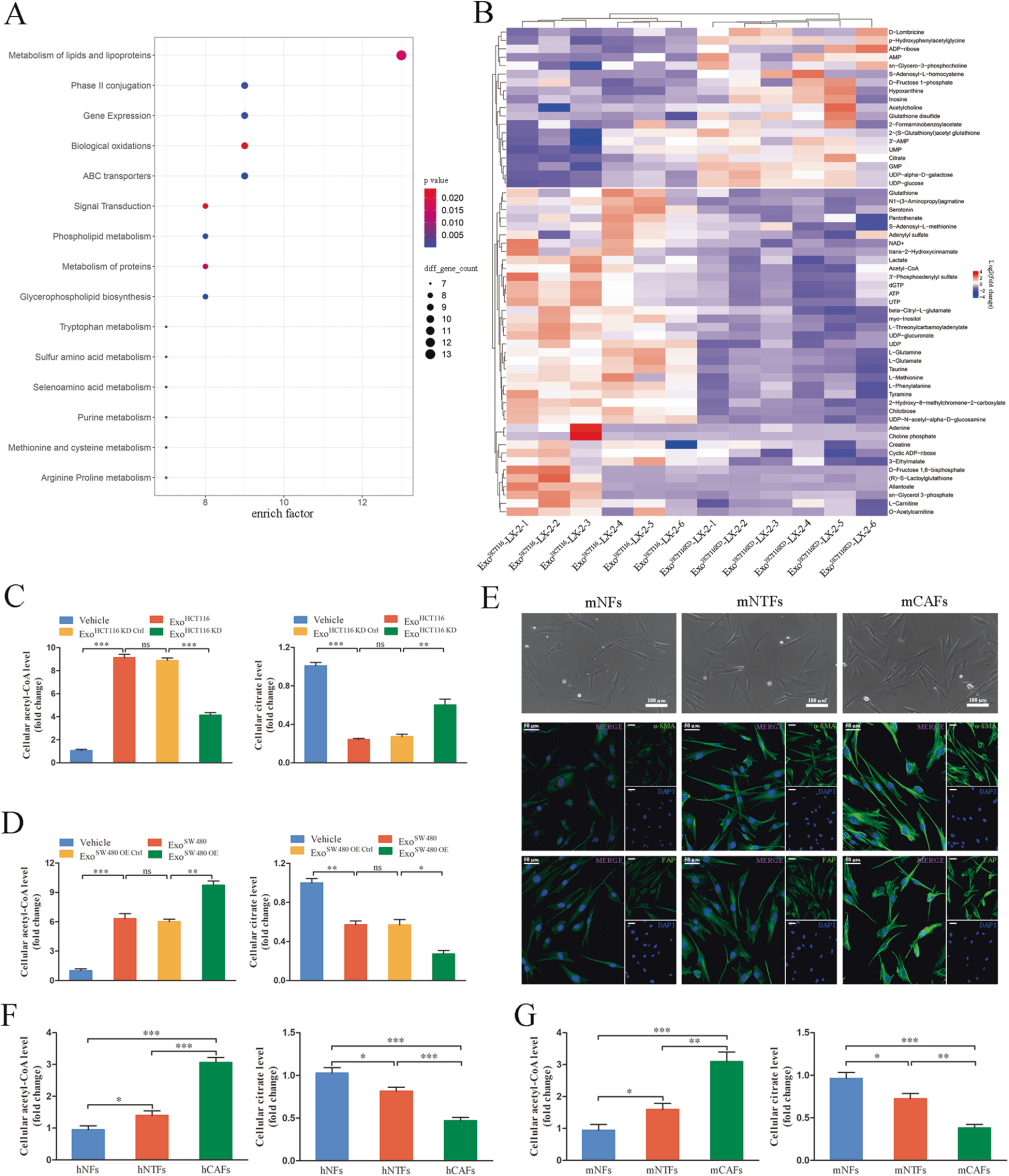
CRC cell-derived exosomal HSPC111 alters lipid metabolism by increasing the level of acetyl-CoA in CAFs. **A** Bubble diagram of KEGG pathway enrichment analysis related to LX-2 cells incubated with Exo^HCT116^, compared with incubated with Exo^HCT116 KD^. **B** Heat map of significantly altered metabolites after HSCs incubated with Exo^HCT116^, compared with incubated with Exo^HCT116 KD^; p value < 0.05 are indicated. **C** Levels of acetyl-CoA and citrate in LX-2 cells incubated with Exo^HCT116 KD^ and their relative control. **D** Levels of acetyl-CoA and citrate in LX-2 cells incubated with Exo^SW480 OE^ and their relative control. **E** Normal fibroblasts (mNFs), non-tumoral fibroblasts (mNTFs) and cancer-associated fibroblasts (mCAFs) isolated from mouse liver tissues were photographed by light micrographs (top). Scale bar = 100 μm. Representative immunofluorescence staining of α-SMA and FAP in mNFs, mNTFs and mCAFs isolated from mouse liver tissues (bottom). Scale bar = 50 μm. Levels of acetyl-CoA and citrate in primary NFs, NTFs and CAFs isolated from human (**F**) and mouse (**G**) liver tissues. Each experiment was performed in triplicate. Data are shown as mean ± SD. *P < 0.05, **P < 0.01, ***P < 0.001.

To further evaluate the effects of exosomal HSPC111 on metabolite changes of CAFs, metabolomic analysis was conducted in Exo^HCT116^ and Exo^HCT116 KD^ incubated LX-2 cells. KEGG pathway enrichment analysis showed that Exo^HCT116^ incubation most markedly influence metabolism of lipids and lipoproteins (Fig. 3A). In our previous studies, we have found that acetyl-CoA metabolism played a critical role in cancer metastasis [23]. Notably, acetyl-CoA level was increased and citrate level was reduced in CAFs incubated with Exo^HCT116^, compared with incubation with Exo^HCT116 KD^ (Fig. 3B and Supplementary Table 6).

Next, we validated whether HSPC111 was involved in regulating acetyl-CoA level. After incubation with Exo^HCT116 KD^, the acetyl-CoA level in CAFs was significantly decreased, whereas the citrate level was significantly increased (Fig. 3C). When treated with Exo^SW480 OE^, the acetyl-CoA level in CAFs was significantly elevated, whereas the citrate level was significantly reduced (Fig. 3D). Subsequently, to evaluate the levels of acetyl-CoA and citrate in primary CAFs, we isolated primary fibroblasts from mouse liver tissues, which exhibited a spindle-like morphology and confirmed by immunofluorescence staining (Fig. 3E and Supplementary Fig. 3B). Next, we examined the levels of acetyl-CoA and citrate in each type of primary fibroblasts, and found that acetyl-CoA level was elevated and citrate level was decreased in mCAFs and mNTFs compared with mNFs (Fig. 3F-G).

**Fig. 4:**
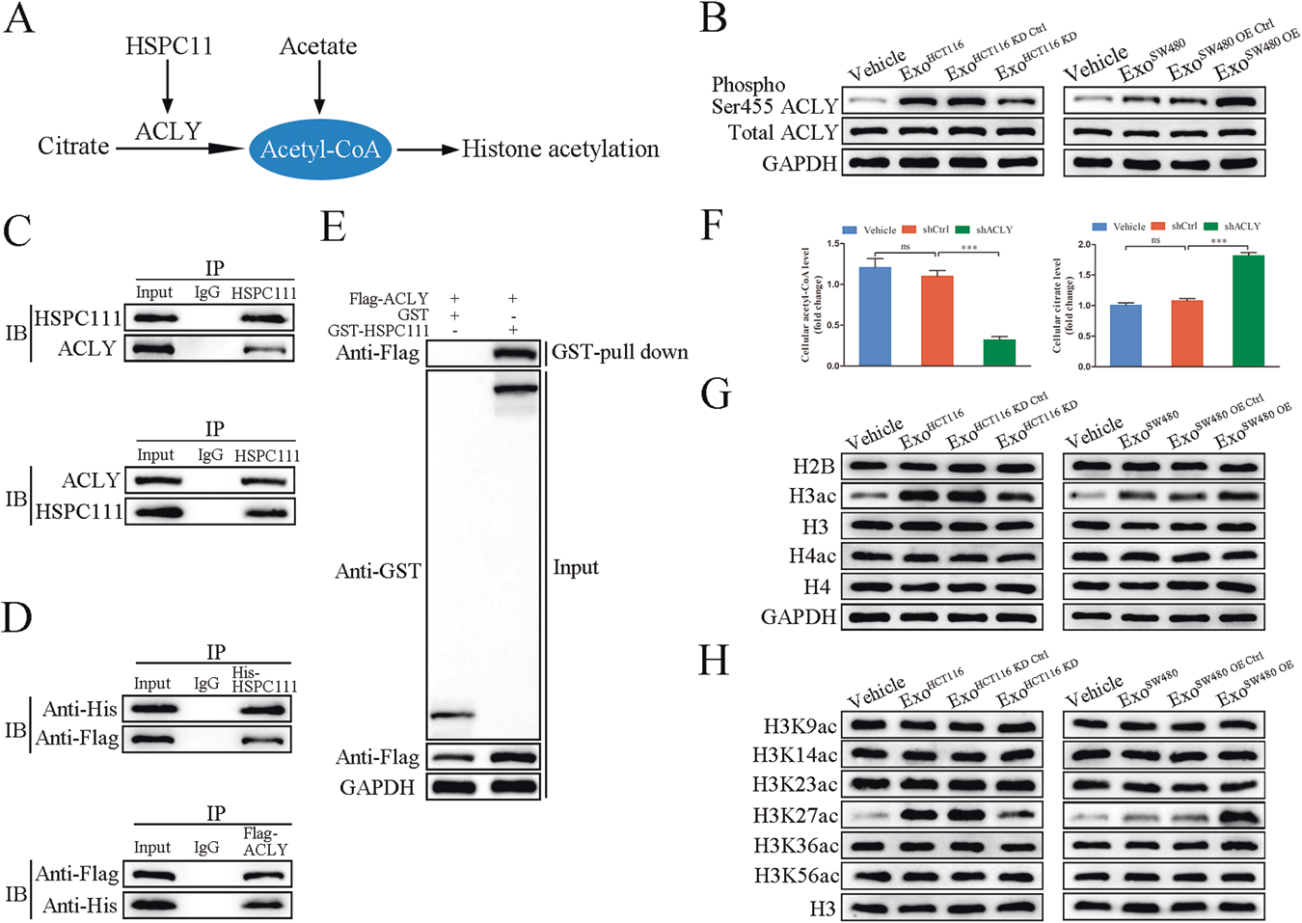
CRC cell-derived exosomal HSPC111 increases phosphorylation level of ACLY and H3K27ac level in CAFs. **A** Diagram of metabolism of acetyl-CoA in CAFs. **B** Western blot showed Ser455 phosphorylation of ACLY in LX-2 cells incubated with Exo^HCT116 KD^ and Exo^SW480 OE^ and their relative control respectively. **C** Co-IP of endogenous HSPC111 and ACLY in LX-2 cells showing co-precipitated ACLY after HSPC111 immunoprecipitation and co-precipitated HSPC111 after ACLY immunoprecipitation. **D** Co-IP of exogenous expressed His-tagged HSPC111 and Flag-tagged ACLY from HEK-293T cells reveals an interaction between HSPC111 and ACLY proteins on western blot. **E** GST pull-down assay. GST or GST-HSPC111 fusion proteins expressed in BL21 cells were purified with glutathione agarose resin and incubated with the lysate of Flag-ACLY-expressing cells. Western blot analysis was performed using anti-GST, anti-Flag and anti-GAPDH. **F** Levels of acetyl-CoA and citrate in LX-2 shACLY cells incubated with Exo^HCT116^. Levels of histone H2B, histone H3 acetylation (H3ac), histone H4 acetylation (H4ac) (**G**) and acetylation of different histone proteins (**H**) in LX-2 cells incubated with Exo^HCT116 KD^ and Exo^SW480 OE^ and their relative control respectively. Each experiment was performed in triplicate. Data are shown as mean ± SD. *P < 0.05, **P < 0.01, ***P < 0.001, ns not significant.

As ATP-citrate lyase (ACLY) activity was regulated at the posttranslational level by phosphorylation [24] (Fig. 4A), we reasoned that the changed cellular acetyl-CoA level might also be the result of elevated phosphorylation level of ACLY. Western blot showed that ACLY phosphorylation was triggered in Exo^SW480 OE^ incubation in CAFs, which was diminished in CAFs incubated with Exo^HCT116 KD^ (Fig. 4B). To further investigate whether HSPC111 and ACLY could directly interact, Co-IP assays revealed the endogenous and exogenous interaction between HSPC111 and ACLY, respectively (Fig. 4C, D). Meanwhile, GST pull-down assays showed GST-fused HSPC111, but not GST, could pull down ACLY (Fig. 4E). Subsequently, to confirm that HSPC111 in exosomes derived from CRC cells affects acetyl-CoA level in LX-2 cells by interacting ACLY, we employed ACLY silencing (shACLY) LX-2 cells (Supplementary Fig. 2C) and found that incubation with Exo^HCT116^ significantly decreased acetyl-CoA level and increased citrate level in shACLY LX-2 cells (Fig. 4F).

Next, as fluctuation of cellular acetyl-CoA levels affects histone acetylation [25] (Fig. 4A), we detected the levels of histone acetylation, and found that incubation with Exo^HCT116 KD^ in CAFs led to a dramatic reduction of histone H3 acetylation (H3ac) level, while incubation with Exo^SW480 OE^ resulted in a significant elevation of H3ac level in CAFs (Fig. 4G). However, a significant change in histone H2B acetylation and histone H4 acetylation (H4ac) levels was not observed (Fig. 4G). Then, we analyzed the levels of histone acetylation at six lysine residues of histone H3 tails by western blot. CAFs incubated with Exo^HCT116 KD^ or Exo^SW480 OE^ led to significant changes in the H3K27 acetylation (H3K27ac) level, but not at other five lysine residues (Fig. 4H). We also detected the general protein acetylation in CAFs incubated with Exo^HCT116 KD^ or Exo^SW480 OE^ and found that a specific band at the position of 17 kDa was altered, which is possibly the band of H3ac (Supplementary Fig. 4A, B). Together, these results suggested that exosomal HSPC111 regulates the acetyl-CoA levels and H3 acetylation by interacting with ACLY in CAFs.

### CRC cell-derived exosomal HSPC111 promotes CAFs to secrete CXCL5

**Fig. 5:**
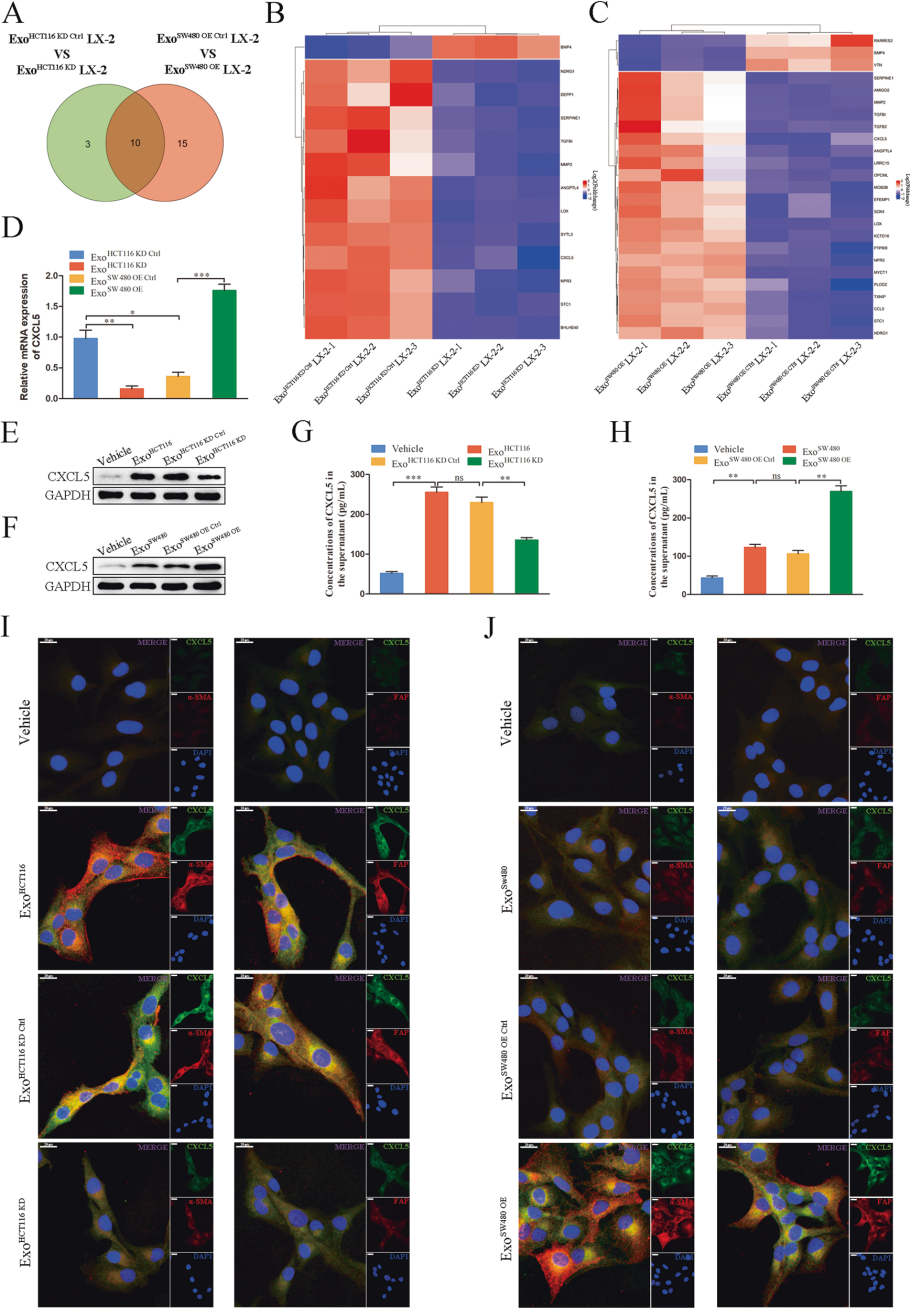
CRC cell-derived exosomal HSPC111 promotes CAFs to secrete CXCL5. A-D LX-2 cells treated with HSPC111-silent and -enriched exosomes were subjected to RNA-seq. Overlapping of genes resulting from comparison of downregulated genes in LX-2 cells incubated with Exo^HCT116 KD^ and upregulated genes in LX-2 cells incubated with Exo^SW480 OE^ (**A**). Heat map of transcriptome alterations after incubating LX-2 cells with Exo^HCT116 KD^ and Exo^HCT116 KD Ctrl^ (**B**). Heat map of transcriptome alterations after incubating LX-2 cells with Exo^SW480 OE^ and Exo^SW480 OE Ctrl^ (**C**). The relative mRNA level of CXCL5 was analyzed in a group of independent samples (**D**). Western blot showed protein level of CXCL5 in LX-2 cells incubated with Exo^HCT116 KD^ (**E**) and Exo^SW480 OE^ (F) and their relative control respectively. ELISA analysis of CXCL5 level in culture medium from LX-2 cells incubated with Exo^HCT116 KD^ (**G**) and Exo^SW480 OE^ (**H**) and their relative control respectively. Immunofluorescence staining of α-SMA, FAP and CXCL5 in LX-2 cells incubated with Exo^HCT116 KD^ (**I**) and Exo^SW480 OE^ (**J**) and their relative control respectively. Scale bar = 20 μm. Each experiment was performed in triplicate. Data are shown as mean ± SD. *P < 0.05, **P < 0.01, ***P < 0.001, ns not significant.

To elucidate the molecular changes elicited by exosomal HSPC111 that promote CRLM, we analyzed global gene expression changes by RNA-seq. By comparing the downregulated genes in CAFs incubated with Exo^HCT116 KD^ (Supplementary Table 7) and upregulated genes in CAFs incubated with Exo^SW480 OE^ (Supplementary Table 8), we observed some cancer-promoting genes, including CXCL5, TGF-β, MMP2, SERPINE1, LOX and STC1 (Fig. 5A–C), suggesting that exosomal HSPC111 may enhance the cancer-promoting effects of HSCs by epigenetic regulation of gene transcription. Since CXCL5 mediates various cellular behaviors including cancer cell migration and metastasis [26], and the CXCL5-CXCR2 axis regulates organ-specific metastasis, including CRLM [27], we selected CXCL5 as a prototype to examine our hypothesis. We first validated the RNA-seq data by qRT-PCR for the selected CXCL5 gene (Fig. 5D). Subsequently, western blot demonstrated that incubation with Exo^HCT116 KD^ significantly decreased CXCL5 protein level in CAFs, whereas incubation with Exo^SW480 OE^ elevated CXCL5 protein level significantly in CAFs (Fig. 5E, F). Furthermore, the concentration of CXCL5 in culture medium was detected by ELISA assay, and the results were consistent with the results of western blot (Fig. 5G, H). Meanwhile, immunofluorescence staining revealed the expression of α-SMA, FAP and CXCL5 was decreased in CAFs incubated with Exo^HCT116 KD^ and increased in CAFs incubated with Exo^SW480 OE^ (Fig. 5I–J). Accordingly, these results indicated that exosomal HSPC111 derived from CRC cells promoted CAFs to produce and release CXCL5.

### An increase in levels of acetyl-CoA and H3K27ac promotes CXCL5 secretion in CAFs

**Fig. 6:**
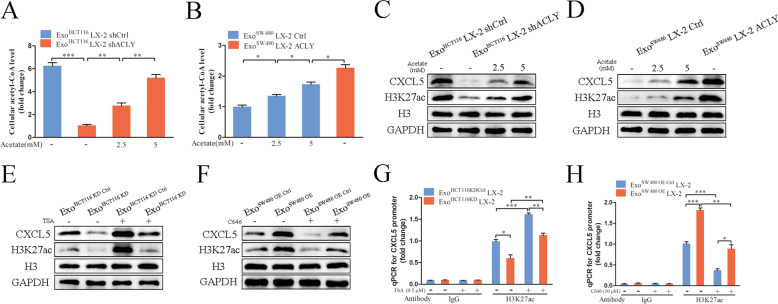
An increase in levels of acetyl-CoA and H3K27ac promotes CXCL5 secretion in CAFs. The effects of acetate treatment on acetyl-CoA levels in LX-2 cells incubated with Exo^HCT116^ (**A**) and Exo^SW480^ (**B**). Western blot showed the effects of acetate treatment on CXCL5 and H3K27ac levels in LX-2 cells incubated with Exo^HCT116^ (**C**) and Exo^SW480^ (**D**). **E** The effects of Trichostatin A (TSA) treatment on CXCL5 and H3K27ac levels in LX-2 cells incubated with Exo^HCT116 KD^ and their relative control. **F** The effects of C646 treatment on CXCL5 and H3K27ac levels in LX-2 cells incubated with Exo^SW480 OE^ and their relative control. **G**, **H** Chromatin immunoprecipitation (ChIP) assays using IgG as a control were performed with antibody against H3K23ac. Examination of H3K27 acetylation status in CXCL5 gene promoter region in LX-2 cells incubated with Exo^HCT116 KD^ and Exo^HCT116 KD Ctrl^ or in LX-2 cells incubated with Exo^HCT116 KD^ and Exo^HCT116 KD Ctrl^ and treated with TSA (**G**). Examination of H3K27 acetylation status in CXCL5 gene promoter region in LX-2 cells incubated with Exo^SW480 OE^ and Exo^SW480 OE Ctrl^ or in LX-2 cells incubated with Exo^SW480 OE^ and Exo^SW480 OE Ctrl^ and treated with C646 (**H**). Each experiment was performed in triplicate. Data are shown as mean ± SD. *P < 0.05, **P < 0.01, ***P < 0.001.

To validate whether the expression of CXCL5 was affected by altering the acetyl-CoA levels, we employed shACLY and ACLY overexpression (ACLY) LX-2 cells (Supplementary Fig. 2D), and found that acetyl-CoA level was decreased in the shACLY group and increased in the ACLY group significantly, compared with control group (Fig. 6A, B). As a supplement of acetate can regulate the acetyl-CoA level [28], we then treated shACLY group and ACLY Ctrl group with different concentration of acetate, and found that acetyl-CoA level was elevated dose-dependently (Fig. 6A, B). Notably, western blot also confirmed that ACLY silencing significantly decreased CXCL5 and H3K27ac protein levels, which increased in ACLY overexpression and acetate treatment group (Fig. 6C, D).

To verify whether HSPC111-dependent alteration of H3K27ac was required for the expression of CXCL5, we treated cells with Trichostatin A (TSA), a histone deacetylase inhibitor, or C646, a p300 acetyltransferase inhibitor, to regulate H3K27ac levels. We found that consistent with the changes of H3K27ac level, TSA treatment significantly enhanced Exo^HCT116 KD Ctrl^ incubation induced CXCL5 protein level, and hindered the effect of Exo^HCT116 KD^ incubation on decreasing CXCL5 protein level in CAFs (Fig. 6E). Meanwhile, C646 treatment hindered the effect of Exo^SW480 OE^ treatment on increasing H3K27ac and CXCL5 protein levels in CAFs (Fig. 6F). Subsequently, ChIP was used to examine histone acetylation on the CXCL5 gene promoter region and found that Exo^HCT116 KD^ incubation decreased H3K27 acetylation on CXCL5 promoter, and TSA increased CXCL5 promoter acetylation in CAFs (Fig. 6G). Additionally, Exo^SW480 OE^ treatment elevated H3K27 acetylation on CXCL5 promoter, and C646 reduced CXCL5 promoter acetylation in CAFs (Fig. 6H). Overall, these results suggested that increases in acetyl-CoA and H3K27ac levels are required for the expression of CXCL5 in CAFs.

### CAFs-derived CXCL5 reinforces exosomal HSPC111 excretion in CRC cells and promotes CRLM progression

**Fig. 7:**
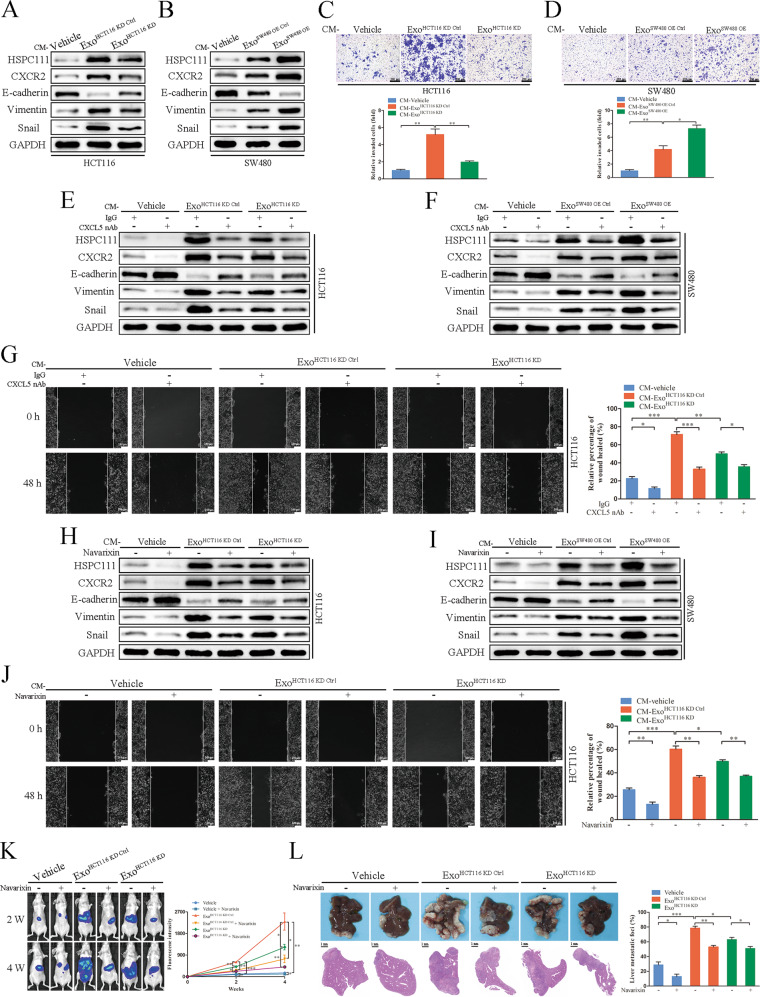
CAFs-derived CXCL5 reinforces exosomal HSPC111 excretion in CRC cells and promotes CRLM progression. Western blot showed the protein levels of HSPC111, CXCR2 and EMT-relative genes in CAFs CM-incubated HCT116 (**A**) and SW480 cells (**B**). Migration of HCT116 (**C**) and SW480 cells (**D**) were analyzed by transwell assay after CAFs CM treatment. Scale bar = 200 μm. Western blot showed the protein levels of HSPC111, CXCR2 and EMT-relative genes in CAFs CM- and CXCL5 neutralizing antibody-incubated HCT116 (**E**) and SW480 cells (**F**). **G** Migration of HCT116 cells were analyzed by wound-healing assay after CAFs CM and CXCL5 neutralizing antibody treatment. Scale bar = 100 μm. Western blot showed the protein levels of HSPC111, CXCR2 and EMT-relative genes in CAFs CM- and CXCR2 inhibitor (navarixin)-incubated HCT116 (**H**) and SW480 cells (**I**). **J** Migration of HCT116 cells were analyzed by wound-healing assay after CAFs CM and CXCR2 inhibitor (navarixin) treatment. Scale bar = 100 μm. **K** IVIS imaging on experimental liver metastasis of indicated mice treated with Exo^HCT116 KD^ or their relative control and with/without navarixin. Luciferase-labeled HCT116 cells were used to perform experimental liver metastasis model (*n* = 3). **L** Representative pictures and quantitative results of HE staining of liver tissue sections from indicated mice. Scale bar = 500 μm. Each experiment was performed in triplicate. Data are shown as mean ± SD. *P < 0.05, **P < 0.01, ***P < 0.001.

To determine whether CAFs-derived CXCL5 exerts feedback regulation on HSPC111 expression in CRC cells. The conditioned medium (CM) from Exo^HCT116 KD^ and Exo^SW480 OE^ treated LX-2 cells was used to incubate HCT116 cells and SW480 cells, respectively. We observed that the protein levels of HSPC111 and CXCR2, a receptor of CXCL5, were decreased in the CM-Exo^HCT116 KD^ incubation group (Fig. 7A) and increased in the CM-Exo^SW480 OE^ incubation group significantly (Fig. 7B). To further explore whether CXCL5 regulates exosomal HSPC111 excretion in CRC cells, we stimulated CRC cells with recombinant human CXCL5, and found that the protein level of HSPC111 was elevated dose-dependently in CRC cells and their derived exosomes, respectively (Supplementary Fig. S5A, B). Additionally, CM-Exo^HCT116 KD^ treated HCT116 cells showed alleviated motility, whereas increased motility was shown in CM-Exo^SW480 OE^ treated SW480 cells (Fig. 7C, D).

To verify whether CXCL5-CXCR2 axis promotes CRC cells migration by inducing EMT, we found that CM-Exo^HCT116 KD^ incubation led to higher expression of E-cadherin and lower expression of Vimentin and Snail in HCT116 cells (Fig. 7A–E). However, downregulation of E-cadherin and upregulation of Vimentin and Snail were observed in SW480 cells incubated with CM-Exo^SW480 OE^ (Fig. 7B–F). Additionally, incubation of a CXCL5-neutralizing antibody with CM-Exo^HCT116 KD^ in HCT116 cells and CM-Exo^SW480 OE^ in SW480 cells not only reduced HSPC111, CXCR2 and E-cadherin expression and elevated Vimentin and Snail expression (Fig. 7E, F), but also hindered the cell migration of CRC cells (Fig. 7G and Supplementary Fig. S5C). Furthermore, consistent with these results, blocking CXCR2 using navarixin, a CXCR2 inhibitor, in CRC cells impaired HSPC111, CXCR2 and EMT-associated genes expression and cell migration of CRC cells as well (Fig. 7H–J and Supplementary Fig. S5D).

To further evaluate whether the CXCL5-CXCR2 axis is involved in CRLM in vivo, we generated a liver metastasis model using nude mice. We found that more metastatic lesions were observed in the Exo^HCT116 KD Ctrl^ group and fewer metastatic lesions were observed in the Exo^HCT116 KD^ group. Notably, nude mice treatment with navarixin inhibited tumor metastasis (Fig. 7K–L), which was also confirmed by histologic analyses. Collectively, these results demonstrated that CXCL5 secreted by CAFs exerts positive feedback on the expression of HSPC111 in CRC cells, resulting in further progression of CRLM.

## Discussion

Tumor microenvironment, a dynamic network orchestrated by intercellular communication, is responsible for cancer progression and metastasis [29]. Therefore, it is necessary to study the interaction between cancer cell and stromal cell mediated by exosomes. Previous studies showed that HSPC111 as a component of ribosomes regulates cell growth [30]. However, how HSPC111 affects cancer metastasis is unknown. In our studies, we showed that CRC cell-derived exosomal HSPC111 converts fibroblasts to CAFs in liver pre-metastatic niche to promote CRLM. Moreover, high expression of HSPC111 in patient’s serum exosomes and primary CRC tissues has a positive correlation with liver metastasis from CRC. Meanwhile, exosomal HSPC111 upregulated acetyl-CoA levels and affected lipid metabolism in CAFs through phosphorylation of ACLY. Recent studies revealed that the elevation of ACLY expression and activity promotes cancer cell growth and metastasis [31, 32], and phosphorylation of ACLY contributes to cellular acetyl-CoA production and then increases histone acetylation [33]. In our study, we observed exosomal HSPC111 treatment specifically affected H3K27 acetylation in CAFs without affecting acetylation of other histone protein.

Tumor cells recruit CAFs to establish a favorable pre-metastatic niche in the stromal microenvironment to support their colonization, growth and invasion in the liver [34, 35]. In recent years, multiple studies have shown that CAFs crosstalk with cancer cells via secreting various cytokines and chemokines [36, 37], and other studies have demonstrated that metabolic reprogramming of CAFs are important in cancer progress [38]. For example, TGF-β1 secreted by CAFs is associated with gastrointestinal stromal tumor migration and metastasis [39]. Moreover, lactate secreted from CAFs could be absorbed by prostate cancer cells and promote their growth [40]. In this study, exosomal HSPC111 derived from CRC cells induces a panel of cancer-promoting factors in CAFs, including CXCL5, TGF-β, MMP2 and SERPINE1, so as to enhance their cancer-promoting effects. Previous studies have elucidated that CXCL5 produced by fibroblasts plays an important role in the progression, growth and metastasis of cancer [41, 42]. Thus, we primarily concentrated on CXCL5 in our study. Since histone modifications have an effect on the gene promoter region, we performed additional experiments and found that TSA or C646 treatment indeed elevated or decreased cellular CXCL5 protein levels, respectively. Taken together, we assumed that CXCL5 may be an important target for inhibiting the pre-metastatic liver microenvironment to suppress CRLM.

We also observed that CM from exosomal HSPC111 incubated CAFs, containing a high concentration of CXCL5, can promote the migration of CRC cells. CXCL5 has been reported to recruit neutrophils in hepatocellular carcinoma to promote tumor growth and metastasis [43]. We pointed out that CXCL5 participated in CAFs-induced CRLM by interaction with CXCR2 in CRC cells and induced EMT phenotype. The CXCL5-CXCR2 axis has been shown to regulate neutrophil homeostasis [44] and facilitate migration and invasion of papillary thyroid carcinoma cells [45] and lung cancer cells [46], but no evidence has been shown that CXCL5 secreted by CAFs is linked with EMT to promote CRLM. In our study, we noticed that CM from exosomal HSPC111 incubated CAFs could downregulate the level of E-cadherin and upregulate the levels of vimentin and snail, which could be abolished by CXCL5 neutralizing antibody. To date, as CXCR2 is the only identified receptor for CXCL5, it is plausible that the pro-metastatic effects of CXCL5 secreted from CAFs will be abolished by inhibiting CXCR2. In the present study, we verified that inhibition of CXCR2 using navarixin was able to reverse the upregulated migration ability and EMT process of CRC cells in vitro, and inhibit CRLM process in vivo. Previous studies demonstrated that once reshaped by cancer cells, stromal cells in the tumor microenvironment exert their regurgitation-feeding activity on cancer cells via secreting cytokines and chemokines [47, 48]. In our study, we found that CXCL5 secreted by CAFs was involved in a feedforward regulatory loop to further increase HSPC111 level in CRC cells, which provide a new perspective on the function of HSPC111 in CRLM progression. These findings may extend our knowledge of the links between CAFs and CXCL5 and shed light on the mechanisms underlying how CAFs facilitate CRLM.

**Fig. 8:**
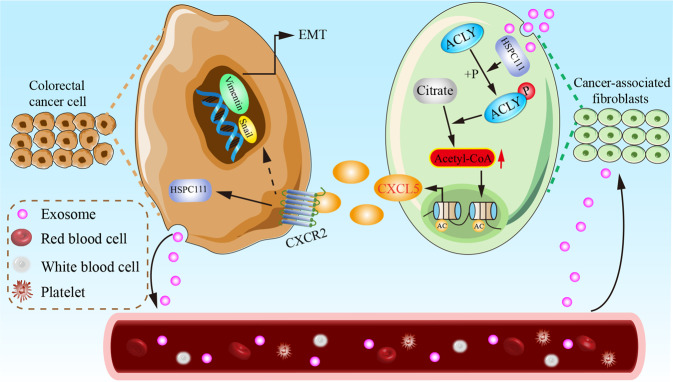
A schematic diagram of mechanism of exosomal HSPC111 facilitates CRLM via reprogramming lipid metabolism in CAFs. Exosomal HSPC111 derived from CRC cells phosphorylates ACLY in CAFs, leading to the increased levels of acetyl-CoA and histone acetylation to secrete CXCL5, and resulting in CRC cells colonized in liver via the CXCL5-CXCR2 axis.

In conclusion, our present results indicate that CRC-derived exosomes function as a critical media for establishing a pre-metastatic niche to promote liver metastasis of CRC cells. Mechanistically, exosomal HSPC111 converts fibroblasts to CAFs and phosphorylates ACLY to increase acetyl-CoA level of CAFs in liver pre-metastatic niche. Meanwhile, increased secretion of CXCL5 from CAFs promotes EMT and metastasis of CRC cells via the CXCL5-CXCR2 axis (Fig. 8). Our study not only demonstrates a novel molecular mechanism of CRLM, but also gives rise to a clinical insight for therapeutic inhibition of HSPC111 and CXCL5-CXCR2 axis to prevent CRLM.

## Supplementary information

Supplementary Figure

Supplementary Table 1

Supplementary Table 2

Supplementary Table 3

Supplementary Table 4

Supplementary Table 5

Supplementary Table 6

Supplementary Table 7

Supplementary Table 8

Checklist

## Data Availability

The data that support the findings of this study are available from the corresponding author upon reasonable request.
