# Supplementary material for: Cancer-derived exosomal HSPC111 promotes colorectal cancer liver metastasis by reprogramming lipid metabolism in cancer-associated fibroblasts
Source: Cell Death Dis. 2022 Jan 13;13(1):57. doi: 10.1038/s41419-022-04506-4 (PMC8758774; doi:10.1038/s41419-022-04506-4)
Supplement: Supplementary file 1 — Supplementary Figure [file 41419_2022_4506_MOESM1_ESM.docx]

**Cancer-derived exosomal HSPC111 promotes colorectal cancer liver metastasis by reprogramming lipid metabolism in cancer-associated fibroblasts**

Chong Zhang^1, 2 *^, Xiang-Yu Wang^1, 2 *^, Peng Zhang^1, 2 *^, Tao-Chen He^1, 2^, Jia-Hao Han^1, 2^, Rui Zhang^1, 2^, Jing Lin^1, 2^, Jie Fan^3^, Lu Lu^1, 2^, Wen-Wei Zhu^1, 2^, Hu-Liang Jia^1, 2^, Ju-Bo Zhang^4^ and Jin-Hong Chen^1, 2 ✉^

^1^Department of General Surgery, Huashan Hospital, Fudan University, 12 Wulumuqi Road (M), Shanghai 200040, China

^2^Institute of Cancer Metastasis, Fudan University, Shanghai, China

^3^Department of Pathology, Huashan Hospital, Fudan University, 12 Wulumuqi Road (M), Shanghai 200040, China

^4^Department of Infectious Diseases, Huashan Hospital, Fudan University, 12 Wulumuqi Road (M), Shanghai 200040, China

^*^These authors contributed equally to this work.

^✉^**Corresponding authors:**

Jin-Hong Chen, MD.

Department of General Surgery, Huashan Hospital, Fudan University, 12 Wulumuqi Road (M), and Institute of Cancer Metastasis, Fudan University, Shanghai 200040, China

Email: jinhongchen@fudan.edu.cn

**Supplementary Figure:**

**Supplementary Figure 1**


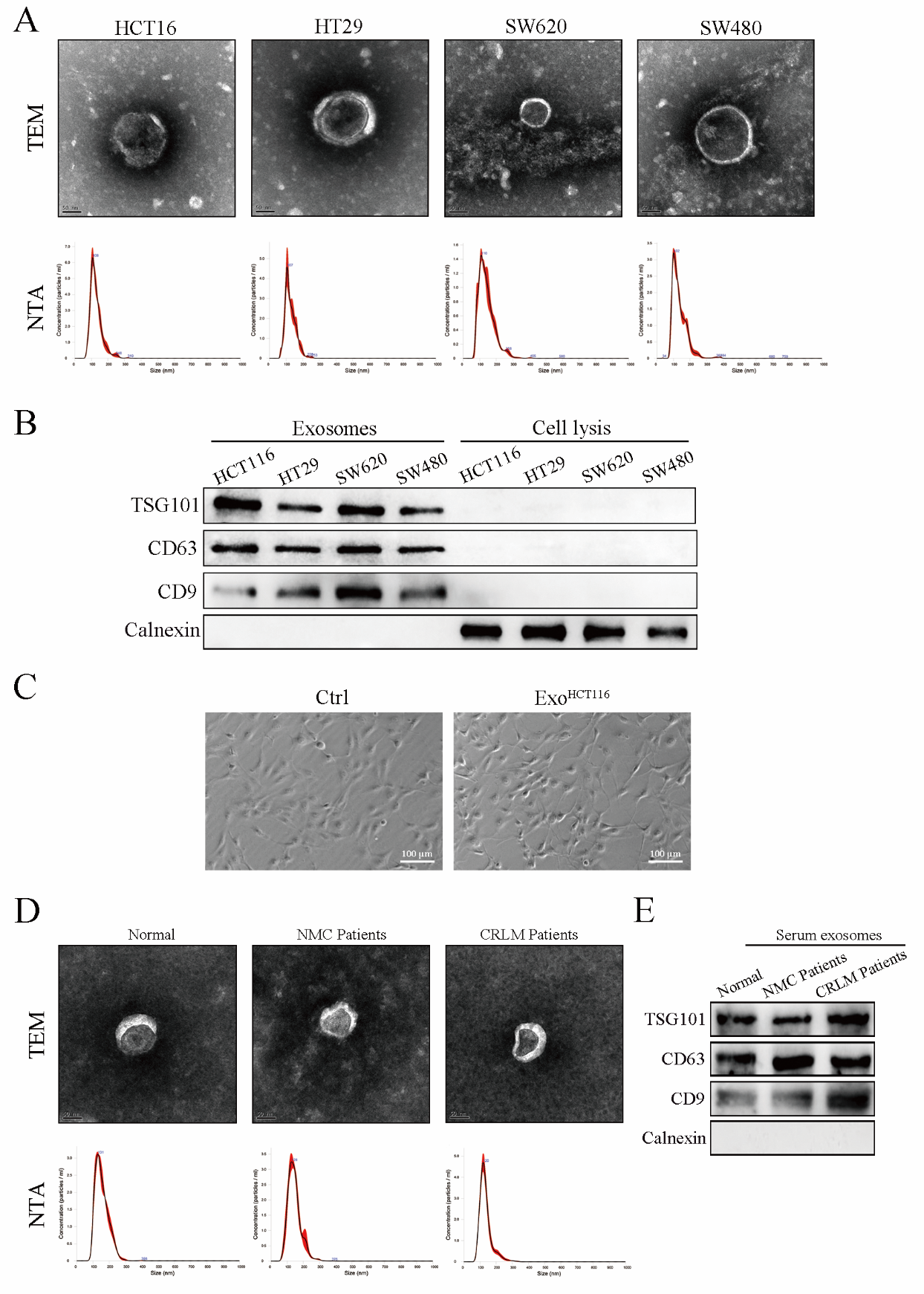


**Supplementary Figure 1. Exosome characterization and morphology of CAFs assays.** **A** Exosomes derived by different CRC cells were determined by transmission electron microscopy (TEM) and Nanosight particle tracking analysis (NTA). Scale bar = 50 nm. **B** Western blot confirmed the positive expression of TSG101, CD63 and CD9 and negative expression of Calnexin in exosomes, and conversely in different CRC cell lines. **C** Morphology of HSCs and CAFs were photographed by light micrographs. Scale bar = 100 μm.. **D** Serum exosomes derived by patients and volunteers were determined by TEM and NTA. Scale bar = 50 nm. **E** Western blot confirmed the positive expression of TSG101, CD63 and CD9 and negative expression of Calnexin in serum exosomes.

**Supplementary Figure 2**


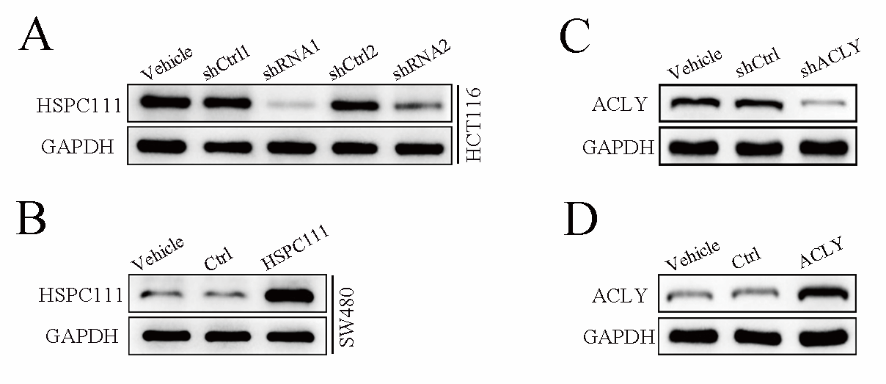


**Supplementary Figure 2. Confirmation of HSPC111 knockdown and overexpression in CRC cells and ACLY knockdown and overexpression in LX-2 cells.** **A-B** Confirmation of the knockdown efficiency of two HSPC111 shRNAs in HCT116 cells (A) and HSPC111 overexpression in SW480 cells (B). **C-D** Confirmation of the knockdown efficiency of ACLY shRNA (C) and ACLY overexpression (D) in LX-2 cells.

**Supplementary Figure 3**


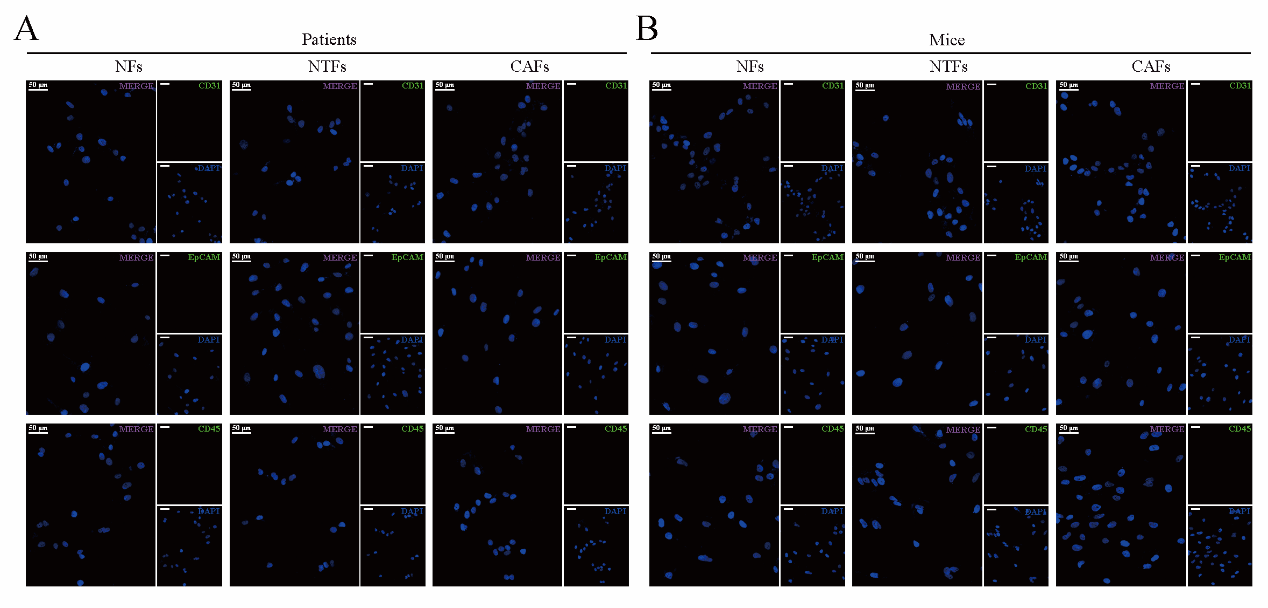


**Supplementary Figure 3. Characterization of NFs, NTFs and CAFs isolated from patient and mouse liver tissues.** **A-B** Representative immunofluorescence staining of CD31, EpCAM and CD45 in NFs, NTFs and CAFs isolated from patient (A) and mouse (B) liver tissues. Scale bar = 50 μm.

**Supplementary Figure 4**


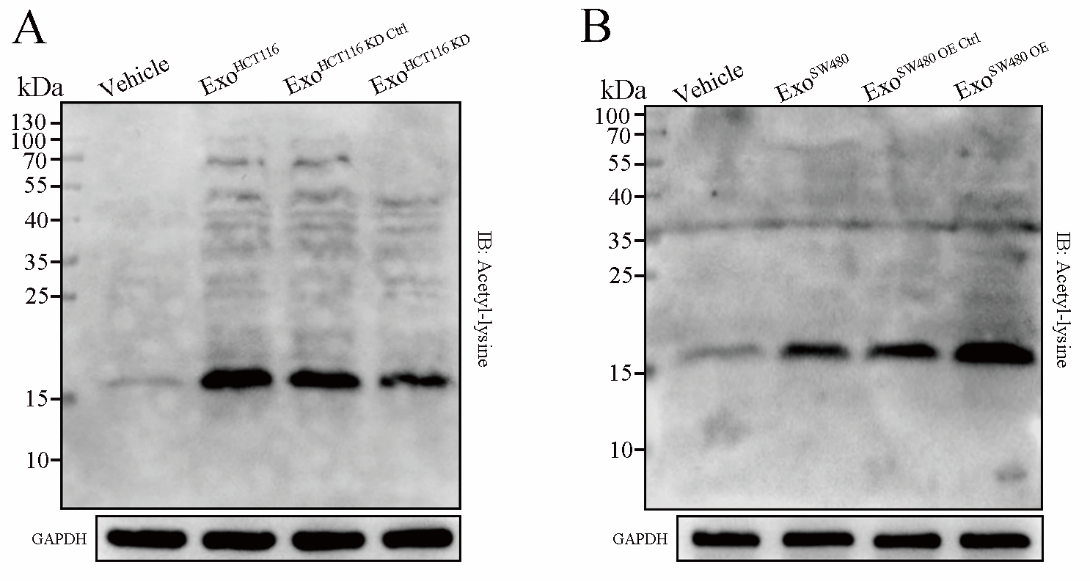


**Supplementary Figure 4. Total protein acetylation assays.** **A-B** The effects of exosomes derived from HSPC111 KD or HSPC111 OE stably expressed HCT116 (A) or SW480 cells (B) on the levels of total protein acetylation in LX-2 cells. Each experiment was performed in triplicate.

**Supplementary Figure 5**


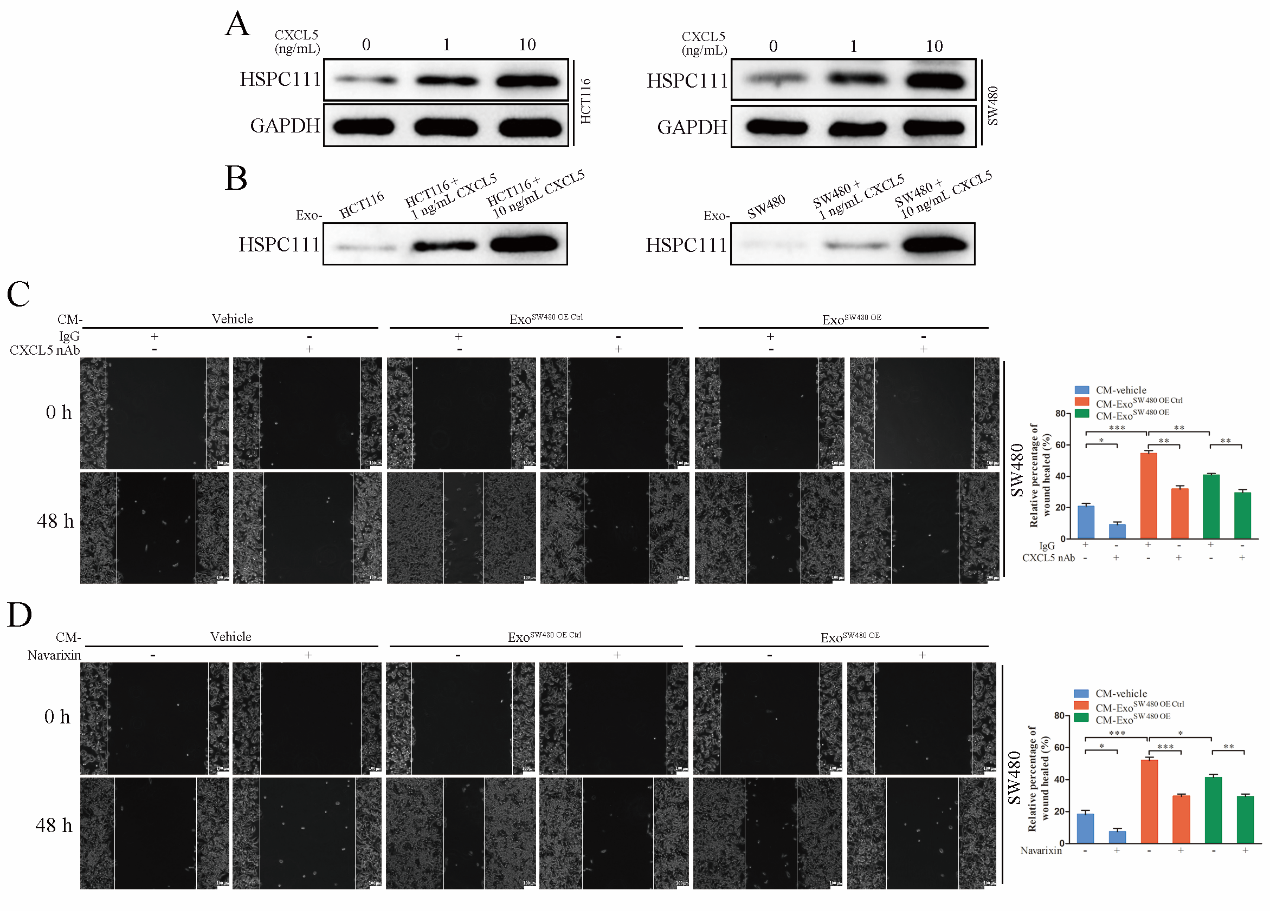


**Supplementary Figure 5. CAFs-derived CXCL5 reinforces exosomal HSPC111 excretion in CRC cells and promotes CRLM progression. Relative to Fig 7.** **A** Western blot showed HSPC111 protein levels in CXCL5 treated HCT116 and SW480 cells. **B** Western blot showed HSPC111 exosomal excretion levels in CXCL5 treated HCT116 and SW480 cells. **C** Migration of SW480 cells were analyzed by wound-healing assay after CAFs CM and CXCL5 neutralizing antibody treatment. Scale bar = 100 μm. **D** Migration of SW480 cells were analyzed by wound-healing assay after CAFs CM and CXCR2 inhibitor (navarixin) treatment. Scale bar = 100 μm. Each experiment was performed in triplicate. Data are shown as mean ± SD. **P* < 0.05, ***P* < 0.01, ****P* < 0.001.
